# Social Media Disorder, Mental Health, and Physical Activity

**DOI:** 10.3390/healthcare14183123

**Published:** 2026-09-21

**Authors:** Abeer F. Almarzouki, Fisal Rajkhan, Khalid A. Alsulami, Ahmad R. Alnoiqy, Mohammed Aljihani, Mohamad Hetta, Omer Shawosh, Yousef Almuqbil, Ibraheem Al Rezqi, Hasan Banaweer, Albasil H. Badawi

**Affiliations:** 1 Department of Clinical Physiology, Faculty of Medicine, King Abdulaziz University, Jeddah 21589, Saudi Arabia; 2 Faculty of Medicine, King Abdulaziz University, Jeddah 21589, Saudi Arabia

**Keywords:** social media disorder, appearance-related anxiety, depression, physical activity, body mass index (BMI), smartphone use

## Abstract

**Background:** Problematic social media use has been associated with effects on mental health, such as greater appearance-related anxiety, which may lead to reduced engagement in physical activity. However, the directions, magnitudes, and mechanisms of the relationships that link social media disorder (SMD), appearance-related anxiety, depressive symptoms, and physical activity remain unclear. This study explored these associations in relation to physical activity and body weight in healthy adults, considering the potential mediating roles of SMD and appearance-related anxiety. **Methods:** Healthy adults completed self-report measures assessing their phone screen time, SMD, appearance-related anxiety (AAI), depressive symptoms, commitment to physical activity (CPAS), physical activity (PA), and body mass index (BMI). We tested the associations among these variables using regression-based models. **Results:** Higher levels of SMD mediated the association between greater phone use and higher levels of appearance-related anxiety, as well as depressive symptoms. A higher BMI and higher levels of camouflaging anxiety were both associated with lower CPAS, which was associated with lower levels of moderate PA. Structural equation modelling revealed that these associations remained significant when adjusting for each variable. **Conclusions:** Overall, the findings of this study support the existence of a network of interconnected relationships between maladaptive social media use, mental health, physical activity, and weight-related outcomes.

## 1. Background

Internet-based platforms, which have become integral to modern daily life, continue to grow [1]. Beyond simple communication, digital engagement influences health behaviours [2], cognitive functioning [3], and mental well-being [4], and a growing body of work has linked higher levels of social media engagement to poorer mental health, including worse depressive and anxiety symptoms, lower self-esteem, and greater self-harm risk [5,6,7,8,9]. Although excessive social media use is not categorised as an addictive disorder in the DSM-5, such behaviour may fulfil the six main criteria for addiction behaviours—namely, mood modification, salience, tolerance, withdrawal symptoms, relapse, and conflict [10]. These concerns motivate continued investigations into the potential mechanisms through which problematic social media use influences mental well-being.

In addition to its mental health correlates, social media exposure may shape both body image and appearance-related concerns [11,12]. Exposure to idealised appearance content and appearance-focused social comparisons on social media has been associated with body dissatisfaction and negative affect, including as a response to ‘fitspiration’ content [12]. Recent evidence in younger populations has also linked appearance-focused social comparison with greater psychological symptom burden [13], while studies in student populations have examined related behavioural correlates, including physical activity and sleep [14]. Appearance-related anxiety, which is characterised by an increased concern about the evaluations of others, may be exacerbated by repeated exposure to idealised body images and online comparisons [15,16].

These observations can be understood through Social Comparison Theory [17], which proposes that individuals evaluate themselves through comparison (e.g., in terms of their appearance) with others. On social media platforms, constant exposure to idealised and unrealistic appearances may shift individuals’ perceptions toward unrealistic virtual standards, creating concerns about how they should look compared with these standards [18]. Such cognitive biases may be particularly relevant to appearance-related anxiety, which includes monitoring, hiding, and avoiding aspects of one’s appearance [19]. In the present study, we therefore considered these dimensions separately rather than treating anxiety as a single undifferentiated construct.

Individuals who experience greater anxiety about their appearance may be more likely to avoid situations in which their bodies are visible or subject to evaluation. This may, in turn, reduce their willingness to engage in physical activity, despite its established associations with beneficial physical and mental health outcomes [20,21,22,23,24]. Appearance concerns may therefore represent one psychological pathway through which weight-related factors are associated with commitment to physical activity; notably, this rationale is consistent with previous research linking appearance and social comparison concerns with body image and behavioural outcomes [25]. On the other hand, depressive symptoms have also been associated with excessive and problematic social media use [26], suggesting that such symptoms are an important factor to consider when examining social media use, physical activity engagement, and subsequent weight-related outcomes. Therefore, the present study explored the associations between SMD, appearance-related anxiety, physical activity commitment, and body mass index (BMI) through the use of an integrated model.

## 2. Methods

### 2.1. Study Design, Population, and Sampling

We conducted a cross-sectional study in Jeddah, Saudi Arabia, between February of 2024 and May of 2025. Adults (≥18 years) residing in Saudi Arabia were recruited via convenience sampling—primarily through local gyms—using a social media announcement (WhatsApp, version 2.24.5.x, Meta Platforms, Inc., Menlo Park, CA, USA). The recruitment announcement was distributed through WhatsApp networks and local gym contacts, and interested individuals accessed the online survey voluntarily. Healthy adults reporting the absence of medical or psychiatric conditions were considered eligible to participate in the study, and a total of 405 participants were enrolled.

### 2.2. Data Collection Methods

Data were collected using self-administered validated questionnaires via Google Forms. The survey included questions on demographic factors (age, sex, marital status, education, and employment), medical conditions and medications, PA and gym attendance, problematic social media use, and mental well-being. BMI was calculated from self-reported height (m) and weight (kg) data. We calculated the internal consistency (Cronbach’s α) for all administered surveys in our sample, and their validity is supported by the surveys’ foundational literature.

#### 2.2.1. Physical Activity and Commitment

Participants reported their gym attendance, weekly frequency, session duration, and activity types (strength/resistance, cardiovascular, stretching/flexibility, HIIT, group classes, aquatic). Physical activity was assessed using the English-language short form of the International Physical Activity Questionnaire (IPAQ-SF), which assesses vigorous activity, moderate activity, walking, and sitting over a 7-day recall period [27]. The IPAQ-SF was scored according to the standard IPAQ scoring protocol, with physical activity expressed as MET-min/week based on the reported frequencies (days/week) and durations (minutes/day) of walking, moderate-intensity, and vigorous-intensity activities [27]. The commitment to PA was measured using the Commitment to Physical Activity Scale (CPAS), which is an 11-item questionnaire rated on a 5-point Likert scale (ranging from strongly disagree to strongly agree) [28].

#### 2.2.2. Phone and Social Media Use

Daily phone screen time (as self-reported from the device’s “screen time” summary over a 7-day period) was recorded, and problematic social media use was assessed using the Social Media Disorder (SMD) scale [29]. The SMD comprises 9 binary (yes/no) items, with total scores ranging from 0 to 9. Following Boer et al. (2022), scores were categorised as normal (≤1), risky (2–5), or problematic (6–9) [30]. Although the SMD-9 was originally developed and validated in adolescents [22], its use has also been examined in adult populations—including young adults aged 18–29 years—with the obtained evidence supporting its reliability and validity in this population [31].

#### 2.2.3. Mental Well-Being and Response Bias

Appearance-related anxiety was assessed using the 10-item Appearance Anxiety Inventory (AAI) developed by Veale et al. (2014) [32], which assesses cognitive and behavioural responses to appearance-related concerns. Each item is rated on a 5-point scale from 0 (not at all) to 4 (all the time), with higher scores indicating greater appearance-related anxiety [32]. The total AAI score is calculated by summing the 10 items, giving a possible score range of 0–40. In the present study, the AAI items were also examined according to three dimensions; namely, threat monitoring, camouflaging, and avoidance. These component scores were calculated from the corresponding AAI items based on the three-factor structure reported by Gumpert et al. (2024) [19] and were examined separately as an exploratory approach to distinguish different cognitive and behavioural responses to appearance concerns. Depressive symptoms were screened using the Patient Health Questionnaire-9 (PHQ-9) [33]. Scores were classified as minimal/no depressive symptoms (0–4), mild (5–9), moderate (10–14), moderately severe (15–19), or severe (20–27) [33]. To account for socially desirable responses, we also implemented the Marlowe–Crowne Social Desirability Scale (MCSDS) [34].

### 2.3. Statistical Analysis

We used linear regression models to assess the associations among daily phone use (hours/day), the SMD score, and the AAI subscale scores (threat monitoring, camouflaging, and avoidance), adjusting for age and sex. When the associations supported an indirect pathway, we tested whether the SMD score mediated the relationship between phone use and appearance anxiety via non-parametric bootstrap confidence intervals (5000 resamples). We then evaluated associations with BMI using linear regression, which included workout frequency, session duration, activity type (binary indicators), CPAS score, SMD score, AAI subscale score, and PHQ-9 score. This model was adjusted for age, sex, and social desirability (MCSDS). We conducted a further mediation analysis to test whether camouflaging anxiety mediated the association between BMI and CPAS. Finally, we tested the associations between CPAS scores and IPAQ-derived activity levels (walking, moderate, and vigorous). To control for multiple comparisons, we report false discovery rate (FDR)-adjusted *p*-values across the AAI subscales and IPAQ activity types, adjusting for the three subscales within each assessment separately. To improve the normality of the data distributions, square root transformations were applied to the IPAQ, SMD, and AAI scores; all other continuous variables were already approximately normally distributed. After transformation, all variables showed acceptable skewness (<1.0) and kurtosis (<1.5). The model assumptions were assessed using residual diagnostics (including QQ plots). Analyses were conducted in R (version 4.5.0), with mediation analyses conducted via the mediation package [35].

Finally, we performed exploratory structural equation modelling (SEM) with maximum likelihood estimation to simultaneously evaluate the associations between phone use, SMD, psychological symptoms, CPAS, PA, and BMI. Variables that were transformed to improve normality (IPAQ, SMD, AAI) were used for modelling. Model fit was assessed according to the χ2 value relative to a null model, the comparative fit index (CFI), the Tucker–Lewis Index (TLI), the root mean square error of approximation (RMSEA), the standardised root mean residual (SRMR), and the χ2/df value. Modelling was carried out using the R package lavaan.

This model was identified using significant associations in regression analyses and is not a confirmatory evaluation of a pre-specified model. Phone use estimates were missing in 13 participants, and BMI and physical activity in one. These individuals were excluded from analyses of their respective variables and from the final SEM.

## 3. Results

### 3.1. Sample Characteristics

#### 3.1.1. Demographics

The sample included 405 participants (69.1% male) with an average age of 26.3 years (SD = 8.5). The mean BMI was 25.8 (SD = 5.1); a total of 33.7% of the participants were classified as overweight, and 16.3% were classified as obese. The participants reported a mean daily phone use of 5.7 h (SD = 2.8). The participants’ characteristics are summarised in Table 1. In this sample, the internal consistency values were found to be acceptable for the SMD (α = 0.79), AAI (α = 0.86), CPAS (α = 0.73), PHQ-9 (α = 0.83), and MCSDS (α = 0.69) scales.

#### 3.1.2. Physical Activity

All participants reported engaging in at least brief regular workouts (Table 2). Resistance training was the most reported activity type, followed by cardiovascular exercise, whereas stretching was the least frequently reported. All observed IPAQ MET-min/week values were below the protocol-based upper limits in each of walking, moderate, and vigorous activity.

#### 3.1.3. Mental Well-Being

Table 3 summarises the questionnaire results. More than half of the participants had SMD scores that fell within the risky range (52.6%), and 21.2% had scores in the problematic range. For depressive symptoms, 21.7% reported minimal symptoms, while most participants reported at least mild depressive symptoms (according to the PHQ-9 classification).

### 3.2. Phone Use, Social Media, and Mental Well-Being

#### 3.2.1. The Role of Appearance Anxiety

Longer daily phone use was associated with higher appearance anxiety scores across the three AAI subscales—avoidance (t(388) = 2.76, *p* = 0.018), camouflaging (t(388) = 2.26, *p* = 0.024), and threat monitoring (t(388) = 2.30, *p* = 0.024). Longer phone use was also associated with higher SMD scores (t(388) = 4.50, *p* < 0.001). Correspondingly, higher SMD scores were associated with greater levels of avoidance (t(401) = 7.36, *p* < 0.001), camouflaging (t(401) = 4.33, *p* < 0.001), and threat monitoring (t(401) = 3.26, *p* = 0.001).

**Figure 1:**
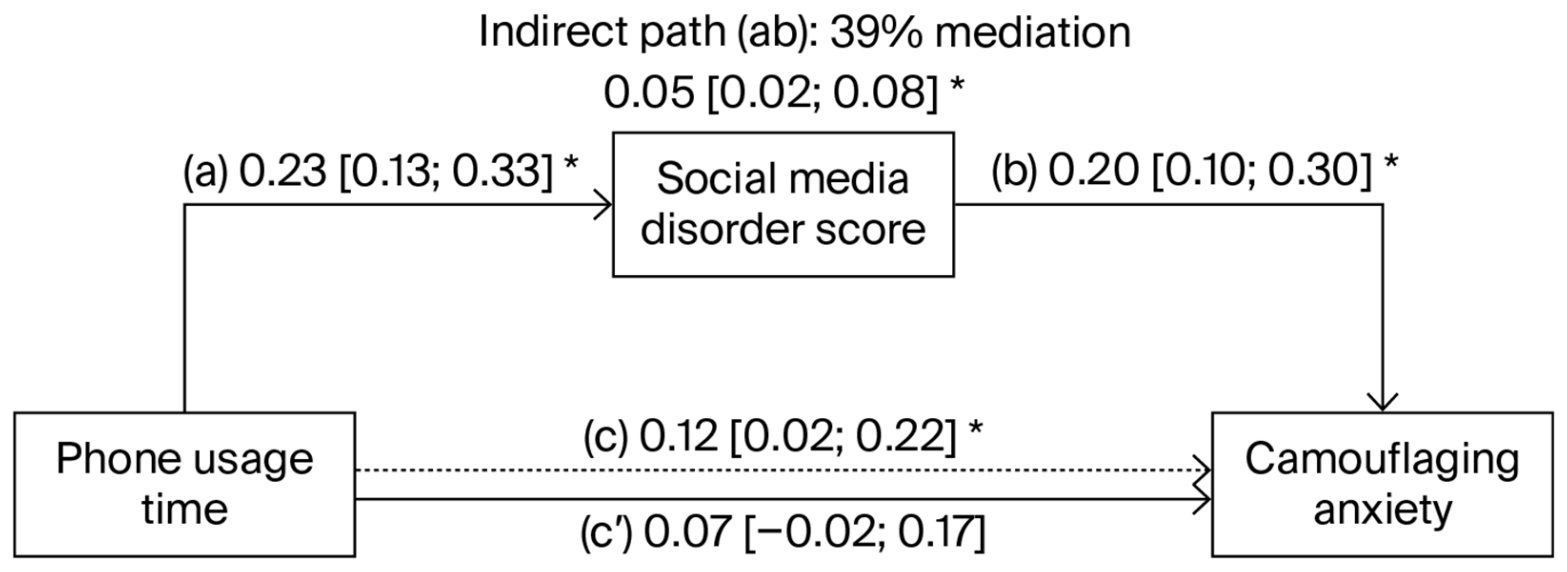
Mediating effect of SMD score in the relationship between daily phone use and camouflaging-related anxiety. Notes: Path (a) shows the association between phone usage time and SMD score; path (b) reveals the association between SMD score and camouflaging anxiety; path (a, b) is the indirect path of the two combined associations with camouflaging anxiety; and path (c) is the association between phone usage time and camouflaging anxiety without adjusting for mediation, while path (c′) refers to the same association after adjusting for mediation. The estimates are displayed with their 95% confidence intervals (CIs), and asterisks (*) denote significance where the 95% CI exceeds 0.

Given this pattern, we tested whether SMD mediated the association between phone use and appearance anxiety. We found that SMD significantly mediated the relationships for avoidance (52% mediated; estimate = 0.03 (0.01, 0.05), *p* < 0.001), camouflaging (39% mediated; estimate = 0.02 (0.01, 0.03), *p* < 0.001), and threat monitoring (29% mediated; estimate = 0.012 (0.002, 0.020), *p* = 0.002). Furthermore, in each model, the direct association between phone use and appearance anxiety was no longer significant after accounting for the indirect effect of SMD. The representative model for camouflaging anxiety is shown in Figure 1.

#### 3.2.2. Role of Depression

Higher PHQ-9 scores were associated with longer phone use (t(388) = 2.88, *p* = 0.004) and higher SMD scores (t(401) = 5.48, *p* < 0.001). Mediation analyses indicated that SMD mediated the association between phone use and depressive symptoms (39% mediated; estimate = 0.13 (0.06, 0.22), *p* < 0.001), with no significant direct association remaining between phone use and the PHQ-9 score after accounting for SMD.

### 3.3. BMI, Physical Activity, Social Media, and Mental Well-Being

**Figure 2:**
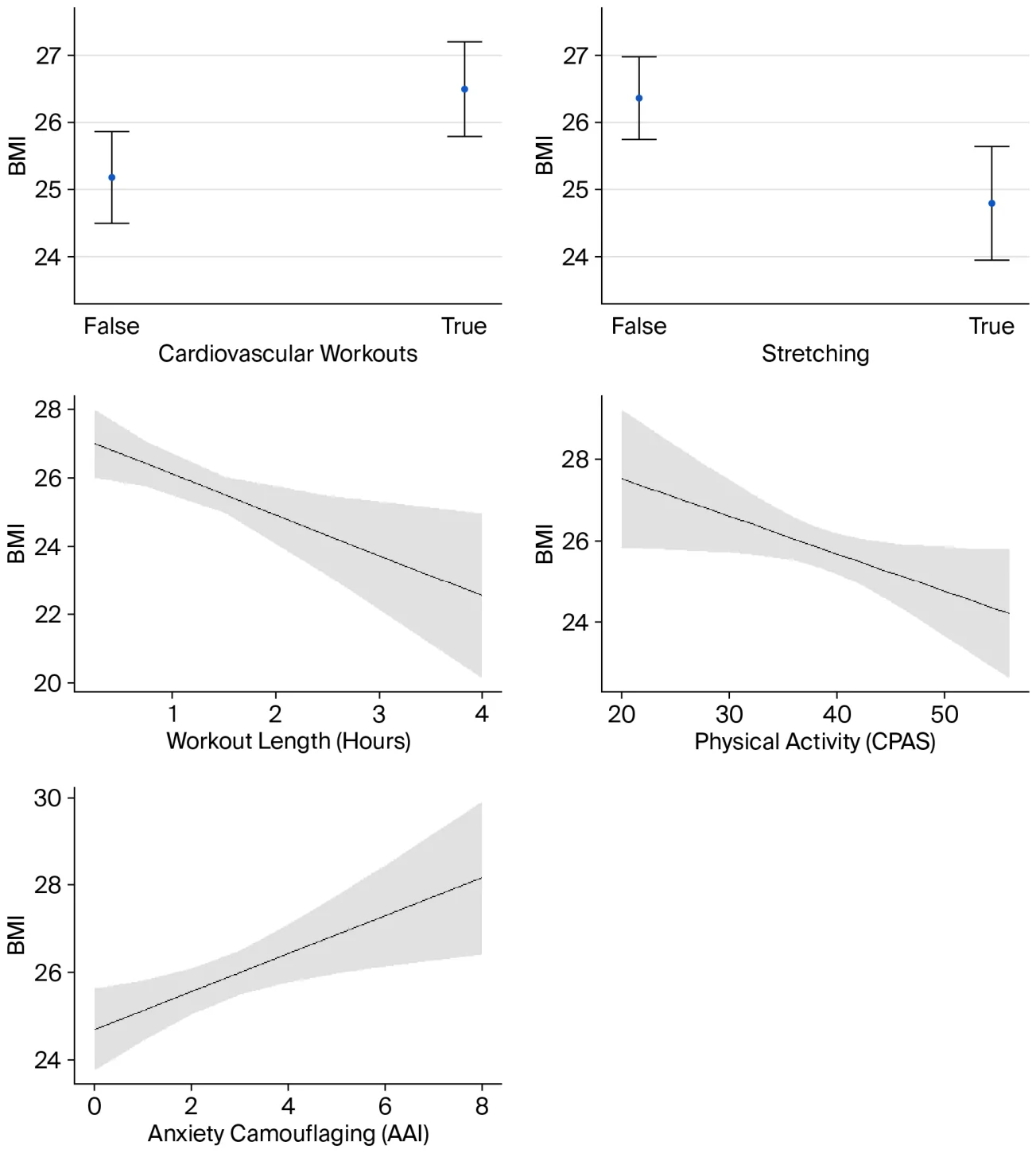
Associations between BMI and greater cardiovascular exercise, less stretching, shorter workouts, lower commitment, and higher camouflaging anxiety. Notes: Statistical testing relies on transformed SMD and AAI scores, while raw scores are depicted for visualization.

Participants with higher BMI were more likely to report cardiovascular workouts (t(384) = 2.64, *p* = 0.008) and less likely to report stretching (t(384) = −2.79, *p* = 0.005). A higher BMI was also associated with shorter workouts (t(384) = −2.76, *p* = 0.006), lower commitment to PA (CPAS; t(384) = −2.03, *p* = 0.043), and higher camouflaging anxiety scores (t(384) = 2.72, *p* = 0.024) (Figure 2). BMI was not significantly associated with depressive symptoms (t(384) = −0.793; *p* = 0.428).

### 3.4. Complete Model of Activity Contributors

Participants with higher BMI reported greater camouflaging-related anxiety and a lower level of PA commitment, and we found that a higher level of camouflaging anxiety was significantly associated with a lower level of PA commitment (t(401) = −3.75, *p* < 0.001). The results of the mediation analysis revealed that camouflaging anxiety was a significant mediator in this relationship (17% mediation, estimate = −0.04 [−0.08, −0.01], *p* < 0.001); however, a significant direct association between a higher BMI and a lower level of PA commitment remained after accounting for this mediator (estimate = −0.20 [−0.34, −0.08], *p* < 0.001). Finally, we found that when we adjusted for the effects of age and sex, a lower level of commitment to PA was significantly associated with less moderate (t(400) = 5.29, *p* < 0.001) and less vigorous (t(400) = 5.23, *p* < 0.001) activity but was not associated with the amount of walking activity.

Three general patterns in the findings emerged from these data. First, phone use is linked to camouflaging anxiety (and other forms of anxiety), with disordered social media use. Second, a higher BMI is associated with a lower commitment to PA—both directly and mediated through camouflaging anxiety. Finally, a lower CPAS is connected to lower activity levels. We further hypothesised that activity levels would be associated with BMI, and thus focused subsequent analyses on camouflaging anxiety specifically, considering its relationships with commitment to PA and BMI.

**Figure 3:**
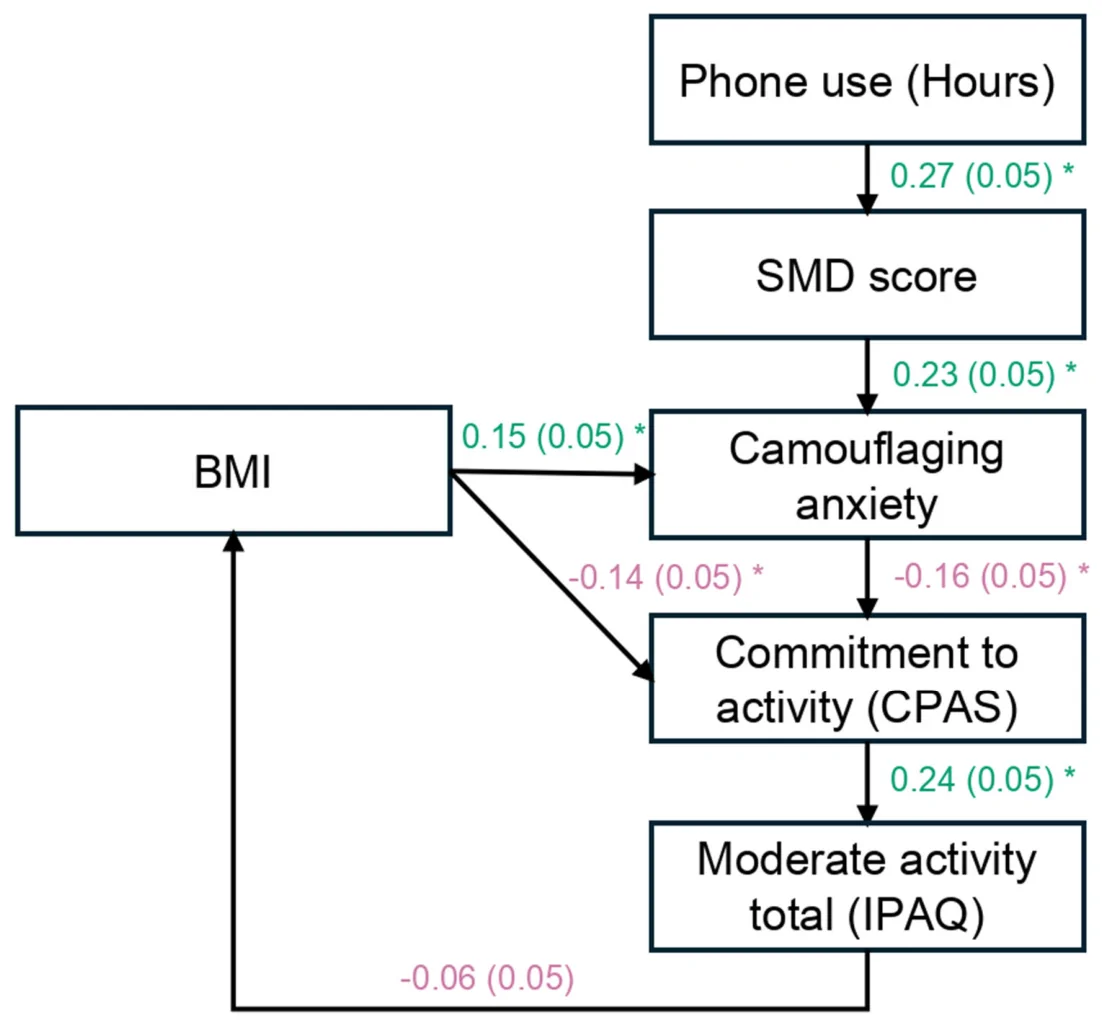
Positive (green) and negative (pink) SEM associations. Standardised coefficients with standard error, with significance marked (*p* < 0.05). Notes: All links in the SEM were significant (*p* < 0.05), except for the amount of moderate activity being associated with BMI (*p* = 0.175). *, *p* < 0.05.

These links are outlined in Figure 3 as standardised coefficients, and were tested using SEM. The model demonstrated an acceptable fit to the data (χ2 = 14.21, DF = 8, *p* = 0.076), comparative fit index (CFI = 0.942), Tucker–Lewis Index (TLI = 0.892), RMSEA (0.045, 90% CI = 0.001, 0.082), SRMR (0.040), and appropriate complexity (χ2/DF = 1.78). The model fit (R2) assessment included SMD (0.073), camouflaging anxiety (0.077), CPAS (0.056), moderate physical activity (0.064), and BMI (0.008).

### 3.5. Sensitivity Analyses

A Monte Carlo simulation (5000 replications, N = 387 participants in the final SEM) was conducted to evaluate the model’s power to detect the reported structural associations. Based on the observed path coefficients and sample size, the estimated power of the significant paths ranged from 0.90 (Camouflaging anxiety > CPAS) to 0.99 (Phone use > SMD). Meanwhile, the non-significant effect of PA on BMI demonstrated low power (0.19) due to its negligible effect size. All significant reported effects exceed the minimum detectable effect size at 0.80 power for 387 individuals (β = 0.14). The Harman’s single-factor test results indicated that the first unrotated factor accounted for 31.8% of the total variance among the SEM variables, suggesting that common method variance is unlikely to account for the observed associations.

## 4. Discussion

We investigated the relationships between screen time, disordered social media use, mental well-being (in terms of self-reported depression symptoms and appearance-related anxiety subscales), PA, and BMI among gym-goers, with the objective of elucidating the network of relationships among these factors. Notably, 73.8% of the sample had SMD scores in the risky or problematic range. Disordered social media use mediated the relationships between screen time and appearance-related anxiety or depressive symptoms. In addition, a higher BMI was associated with higher camouflaging anxiety and lower CPAS values, while a lower CPAS value was associated with lower levels of moderate and vigorous PA. Together, these findings suggest a set of associations through which problematic social media use is linked to worse mental well-being, which is in turn related to lower PA commitment and lower levels of physical activity.

The SMD score was found to mediate the relationships between screen time and both appearance-related anxiety and depressive symptoms, suggesting that problematic social media engagement may be more informative than screen time alone for the examination of unfavourable mental and cognitive outcomes and addictive tendencies [25,36,37]. In previous experimental work, we reported that participants who spent time scrolling on social media had worse working memory performance than those who spent the same amount of time performing other activities on the internet, particularly among those who were at least moderately depressed [38].

The high proportion of participants that scored within risky or problematic ranges is consistent with that in other reports [39], and may reflect an addictive pattern of behaviour that has been described in a related longitudinal work [40]. From a policy perspective, although screen time is often highlighted as a target, the findings suggest that greater attention to social media exposure—rather than overall screen time alone—may be relevant to mental health, which has also been documented previously [41]. In terms of mechanisms, social media platforms frequently promote idealised body images (‘fitspiration’) that may be unrealistic [42], which has been associated with poor body satisfaction [43]. Social media can also disseminate inaccurate health information and promote products that imply unrealistic results [44], and attempts to change oneself based on unrealistic expectations may contribute to distress and frustration [45].

This framework is consistent with our finding that higher SMD scores are associated with higher levels of depressive symptoms and appearance-related anxiety, as well as with prior evidence linking appearance-related social media engagement and preoccupation to increased risks of depression and appearance anxiety [46]. In a large-scale survey of Chinese adolescents, depressive symptoms mediated the association between social media dependence and physical activity [47]. These findings suggest that the relationships between mental well-being, disordered social media use, and physical activity are far more complex than previously understood, and that multiple factors may play a role in shaping these relationships. The relatively high proportion of participants reporting at least mild depressive symptoms (78.3%) in this study should be interpreted in the context of the PHQ-9 being a self-reported measure of depressive symptom severity, rather than a clinical diagnostic cutoff tool. Therefore, the observed prevalence should be considered an indication of potential elevated depressive symptom burden in gym-goers, rather than evidence of elevated rates of clinical depression. Further research involving non-gym-goers will be required to determine whether the levels of depressive symptoms are higher in gym-goers than in the general population.

In our sample, a higher BMI was associated with greater camouflaging anxiety and lower CPAS scores but not with the other appearance anxiety subscales or depressive symptoms. Although the relationship between BMI and psychological comorbidity is well established, it remains heterogeneous across constructs and populations [48,49], and prior studies have suggested that both low and high BMI may be related to elevated anxiety [50]. Conceptually, appearance-related anxiety is more closely tied to body image perceptions than general anxiety symptoms [51], which may explain why BMI was specifically associated with camouflaging anxiety in our study. Related work has reported that BMI influences self-presentation behaviours on Instagram [52], consistent with camouflaging tendencies, i.e., the masking or hiding of perceived flaws. This phenomenon may lead to lower participation in physical activity (lower CPAS and fewer workout hours) among those with a high BMI, or to a focus on exercise aimed at weight loss (e.g., cardiovascular exercise without stretching), as observed in our study. Interestingly, a negative body-related effect may influence exercise patterns independently of BMI; for example, a previous study involving 1200 female participants demonstrated that body shame is associated with poor exercise profiles regardless of BMI [53]. Having a more positive body image or perception (or less body shame) was associated with more balanced exercise routines and better dietary habits [53]. In our gym-going sample, this point is particularly relevant because our recruitment methods likely led to the underrepresentation of individuals with higher BMI, who may avoid gym settings. This restricted range may have reduced the magnitude of the reported association, yet the relationship remained significant. Thus, the associations observed here may differ from those observed in broader community-based samples with a broader BMI distribution.

There are several limitations that are important to consider when interpreting these findings. Our design was cross-sectional, which precludes conclusions related to temporal ordering and causality. While we modelled a feedback loop between BMI and PA to test their cross-sectional associations, a longitudinal design would more fully elucidate these relationships. The purpose of our SEM analysis was to assess whether the associations identified in preliminary analyses held when estimated concurrently; however, this model was exploratory rather than confirmatory of a pre-specified structure. In addition, the convenience sampling strategy may have limited the results’ generalisability and introduced selection bias. Participants were recruited primarily through local gyms and social media announcements, which may have biased enrolment towards individuals who were more physically active and more engaged with social media. This may have affected the observed prevalence of SMD and depressive symptoms and limits the extent to which these findings can be generalised to the broader adult population. Key variables were self-reported—including BMI, which was derived from participants’ self-reported height and weight, and phone use, which was calculated based on reports of their devices’ screen time summaries—which may have introduced measurement errors. In addition, although the IPAQ was originally validated across populations with a broad range of physical activity levels [27], its application to a narrower population of gym-goers may introduce certain limitations, particularly given the potential for differences in reporting of vigorous physical activity. Therefore, the IPAQ findings should be interpreted with consideration of the characteristics of this specific sample. Furthermore, we did not assess content-specific exposure (e.g., ‘fitspiration’ or appearance-focused influencers), which could help to further clarify the links between disordered social media use, appearance anxiety, and PA behaviours [54]. Future research may also benefit from an examination of other contributors to appearance anxiety (e.g., body-shaming from peers or family), which can shape avoidance and anxiety-related behaviours [55].

## 5. Conclusions

Overall, the presented findings suggest an interconnected pattern of associations between problematic social media use, mental well-being, physical activity commitment, and BMI among study participants. These findings may be relevant to public health, highlighting problematic social media use and appearance-related concerns as factors that could be considered when assessing mental well-being and physical activity engagement. However, given the convenience sampling design and the specific characteristics of the gym-going sample, this study’s findings require replication in a broader healthy adult population. Furthermore, the present cross-sectional findings do not allow for the assessment of causal or intervention effects. Future prospective and intervention-based research is needed to determine the directionality of these associations and whether addressing problematic social media use or appearance-related concerns could improve physical activity and related health outcomes.

## Figures and Tables

**Table 1:** Sociodemographic and anthropometric characteristics of the study participants.

Characteristic	Value
Sample size	405
Age, mean (SD)	26.33 (8.45)
Sex: male, N (%)	280 (69.1)
BMI, mean (SD)	25.82 (5.14)
BMI group, N (%)	
Normal	202 (50.0)
Overweight	136 (33.7)
Obese	66 (16.3)
Education, N (%)	
High school/intermediate	80 (19.7)
University	306 (75.6)
Postgraduate	19 (4.7)
Employment, N (%)	
Not employed	37 (9.1)
Student	243 (60.0)
Employed	125 (30.9)
Daily phone use hours, mean (SD)	5.69 (2.75)

**Table 2:** Physical activity frequency, duration, and type reported by the study participants.

Measure	Value
Workouts per week, mean (SD)	3.6 (1.6)
Workout length, N (%)	
<30 min	50 (12.3)
30 min–1 h	115 (28.4)
1–2 h	197 (48.6)
2–3 h	39 (9.6)
>3 h	4 (1.0)
Workout type, N (%)	
Strength/resistance	308 (76.0)
Cardiovascular	195 (48.1)
Stretching	141 (34.8)
High-intensity interval	71 (17.5)
Group classes	59 (14.6)
Aquatic	50 (12.3)
IPAQ activity, mean (SD)	
Walking MET-min/week	624.67 (660.89)
Moderate MET-min/week	678.66 (682.43)
Vigorous MET-min/week	1050.59 (1219.03)
Total MET-min/week	2353.93 (1929.87)

**Table 3:** Mental well-being questionnaire results for the study participants.

Measure	Mean (SD)
Commitment to physical activity (CPAS), mean (SD)	38.61 (6.61)
Social desirability (MCSDS), mean (SD)	8.25 (2.47)
Social media disorder (SMD), N (%)	
Normal	106 (26.2)
Risky	213 (52.6)
Problematic	86 (21.2)
Appearance anxiety (AAI), mean (SD)	14.24 (8.67)
Threat monitoring (AAI), mean (SD)	8.40 (4.37)
Camouflaging (AAI), mean (SD)	2.58 (2.37)
Avoidance (AAI), mean (SD)	3.26 (3.18)
Depressive symptoms (PHQ-9), N (%)	
Minimal	88 (21.7)
Mild	119 (29.4)
Moderate	100 (24.7)
Moderately severe	73 (18.0)
Severe	25 (6.2)

## Data Availability

The data generated and analyzed during this study are not publicly available due to privacy and ethical restrictions, as they contain information collected from human participants. The data may be made available in a de-identified and appropriately restricted form upon reasonable request, subject to approval by the relevant ethics committee and applicable institutional and privacy requirements.

## References

[1] Firth J., Torous J., López-Gil J.F., Linardon J., Milton A., Lambert J., Smith L., Jarić I., Fabian H., Vancampfort D. (2024). From “online brains” to “online lives”: Understanding the individualized impacts of Internet use across psychological, cognitive and social dimensions. World Psychiatry.

[2] Paul B., Headley-Johnson S.-A. (2025). The Impact of Social Media on Health Behaviors, a Systematic Review. Healthcare.

[3] Shanmugasundaram M., Tamilarasu A. (2023). The impact of digital technology, social media, and artificial intelligence on cognitive functions: A review. Front. Cogn..

[4] Ulvi O., Karamehic-Muratovic A., Baghbanzadeh M., Bashir A., Smith J., Haque U. (2022). Social Media Use and Mental Health: A Global Analysis. Epidemiologia.

[5] Behera N., Khuntia S., Pandey K., Shankar S. (2025). Impact of Social Media Use on Physical, Mental, Social, and Emotional Health, Sleep Quality, Body Image, and Mood: Evidence from 21 Countries-A Systematic Literature Review with Narrative Synthesis. Int. J. Behav. Med..

[6] Chen Z., Liao X., Yang J., Tian Y., Peng K., Liu X., Li Y. (2024). Association of screen-based activities and risk of self-harm and suicidal behaviors among young people: A systematic review and meta-analysis of longitudinal studies. Psychiatry Res..

[7] Jing Z., Yang W., Lei Z., Junmei W., Hui L., Tianmin Z. (2025). Correlations between social media addiction and anxiety, depression, FoMO, loneliness and self-esteem among students: A systematic review and meta-analysis. PLoS ONE.

[8] Liu M., Kamper-DeMarco K.E., Zhang J., Xiao J., Dong D., Xue P. (2022). Time Spent on Social Media and Risk of Depression in Adolescents: A Dose-Response Meta-Analysis. Int. J. Env. Res. Public Health.

[9] Ranabhat C.L., Marion J.W., Jakovljevic M. (2025). Association Between Social Media Use and Self-Reported Anxiety and/or Depression: Results from 113 Countries. Int. J. Soc. Psychiatry.

[10] Griffiths M.D. (2013). Social Networking Addiction: Emerging Themes and Issues. J. Addctn. Res. Ther..

[11] Czubaj N., Szymańska M., Nowak B., Grajek M. (2025). The Impact of Social Media on Body Image Perception in Young People. Nutrients.

[12] Prichard I., Kavanagh E., Mulgrew K.E., Lim M.S.C., Tiggemann M. (2020). The effect of Instagram #fitspiration images on young women’s mood, body image, and exercise behaviour. Body Image.

[13] Pedrollo L.F.S., Miasso A.I., Molina N.P.F.M., Di Donato G., Vedana K.G.G. (2025). Depression, Anxiety, Stress, and Dissatisfaction With Life and Appearance During Social Media Use: A Study With Brazilian Graduate Students. Ment. Illn..

[14] Catling J., Hussain I., Khalil A. (2025). Psychological and Behavioural Factors That Impact on the Mental Health of University Students. Ment. Illn..

[15] Putri B.B.K., Noer A.H., Purba F.D. (2025). Psychological Factors Influencing Appearance Anxiety Among Adolescents: A Systematic Literature Review. Psychol. Res. Behav. Manag..

[16] Yao L.S., Niu G.F., Sun X.J. (2024). A longitudinal study on the relationships between social media ideals exposure and thin-ideal internalization, social appearance anxiety, and cosmetic surgery consideration. Body Image.

[17] Myers T.A., Crowther J.H. (2009). Social comparison as a predictor of body dissatisfaction: A meta-analytic review. J. Abnorm. Psychol..

[18] Fardouly J., Diedrichs P.C., Vartanian L.R., Halliwell E. (2015). Social comparisons on social media: The impact of Facebook on young women’s body image concerns and mood. Body Image.

[19] Gumpert M., Rautio D., Monzani B., Jassi A., Krebs G., Fernández de la Cruz L., Mataix-Cols D., Jansson-Fröjmark M. (2024). Psychometric evaluation of the appearance anxiety inventory in adolescents with body dysmorphic disorder. Cogn. Behav. Ther..

[20] Papapanou T.K., Darviri C., Kanaka-Gantenbein C., Tigani X., Michou M., Vlachakis D., Chrousos G.P., Bacopoulou F. (2023). Strong Correlations between Social Appearance Anxiety, Use of Social Media, and Feelings of Loneliness in Adolescents and Young Adults. Int. J. Env. Res. Public Health.

[21] Anderson E., Durstine J.L. (2019). Physical activity, exercise, and chronic diseases: A brief review. Sports Med. Health Sci..

[22] Singh B., Olds T., Curtis R., Dumuid D., Virgara R., Watson A., Szeto K., O’Connor E., Ferguson T., Eglitis E. (2023). Effectiveness of physical activity interventions for improving depression, anxiety and distress: An overview of systematic reviews. Br. J. Sports Med..

[23] Biçki D., Göktaş A., Özkoçak G., Demircioğlu D.T. (2025). Exploring the nexus: Physical activity, body image, and anxiety among university students. BMC Psychol..

[24] Sani S.H.Z., Fathirezaie Z., Brand S., Pühse U., Holsboer-Trachsler E., Gerber M., Talepasand S. (2016). Physical activity and self-esteem: Testing direct and indirect relationships associated with psychological and physical mechanisms. Neuropsychiatr. Dis. Treat..

[25] Zhang Y., Hao J., Guo K., Huang Q., Liu L. (2026). Appearance anxiety and college students’ exercise participation: A chain mediation model of negative body image and exercise self-efficacy. Front. Psychol..

[26] Yuan C., Zhou H., Wang A., Jiang H., Xiao N. (2026). Associations Between Depression and Problematic Social Media Use: A Longitudinal Study and Daily Diary Study. Int. J. Psychol..

[27] Craig C.L., Marshall A.L., Sjöström M., Bauman A.E., Booth M.L., Ainsworth B.E., Pratt M., Ekelund U., Yngve A., Sallis J.F. (2003). International physical activity questionnaire: 12-country reliability and validity. Med. Sci. Sports Exerc..

[28] Debate R.D., Huberty J., Pettee K. (2009). Psychometric properties of the commitment to physical activity scale. Am. J. Health Behav..

[29] van den Eijnden R.J.J.M., Lemmens J.S., Valkenburg P.M. (2016). The Social Media Disorder Scale. Comput. Hum. Behav..

[30] Boer M., Stevens G.W.J.M., Finkenauer C., Koning I.M., van den Eijnden R.J.J.M. (2022). Validation of the Social Media Disorder Scale in Adolescents: Findings From a Large-Scale Nationally Representative Sample. Assessment.

[31] Kokka I., Mourikis I., Michou M., Vlachakis D., Darviri C., Zervas I., Kanaka-Gantenbein C., Bacopoulou F. (2021). Validation of the Greek Version of Social Media Disorder Scale. Adv. Exp. Med. Biol..

[32] Veale D., Eshkevari E., Kanakam N., Ellison N., Costa A., Werner T. (2014). The Appearance Anxiety Inventory: Validation of a Process Measure in the Treatment of Body Dysmorphic Disorder. Behav. Cogn. Psychother..

[33] Kroenke K., Spitzer R.L., Williams J.B. (2001). The PHQ-9: Validity of a brief depression severity measure. J. Gen. Intern. Med..

[34] O’Grady K.E. (1988). The Marlowe-Crowne and Edwards Social Desirability Scales: A Psychometric Perspective. Multivar. Behav. Res..

[35] Tingley D., Yamamoto T., Hirose K., Keele L., Imai K. (2014). mediation: R Package for Causal Mediation Analysis. J. Stat. Softw..

[36] Shannon H., Bush K., Shvetz C., Paquin V., Morency J., Hellemans K.G.C., Guimond S. (2024). Longitudinal Problematic Social Media Use in Students and Its Association with Negative Mental Health Outcomes. Psychol. Res. Behav. Manag..

[37] Tsilosani A., Chan K., Steffens A., Bolton T.B., Kowalczyk W.J. (2023). Problematic social media use is associated with depression and similar to behavioral addictions: Physiological and behavioral evidence. Addict. Behav..

[38] Almarzouki A.F., Alghamdi R.A., Nassar R., Aljohani R.R., Nasser A., Bawadood M., Almalki R.H. (2022). Social Media Usage, Working Memory, and Depression: An Experimental Investigation among University Students. Behav. Sci..

[39] Alfaya M.A., Abdullah N.S., Alshahrani N.Z., Alqahtani A.A.A., Algethami M.R., Al Qahtani A.S.Y., Aljunaid M.A., Alharbi F.T.G. (2023). Prevalence and Determinants of Social Media Addiction among Medical Students in a Selected University in Saudi Arabia: A Cross-Sectional Study. Healthcare.

[40] Hernández C., Ferrada M., Ciarrochi J., Quevedo S., Garcés J.A., Hansen R., Sahdra B. (2024). The cycle of solitude and avoidance: A daily life evaluation of the relationship between internet addiction and symptoms of social anxiety. Front. Psychol..

[41] Sanzari C.M., Gorrell S., Anderson L.M., Reilly E.E., Niemiec M.A., Orloff N.C., Anderson D.A., Hormes J.M. (2023). The impact of social media use on body image and disordered eating behaviors: Content matters more than duration of exposure. Eat. Behav..

[42] Aparicio-Martinez P., Perea-Moreno A.J., Martinez-Jimenez M.P., Redel-Macías M.D., Pagliari C., Vaquero-Abellan M. (2019). Social Media, Thin-Ideal, Body Dissatisfaction and Disordered Eating Attitudes: An Exploratory Analysis. Int. J. Environ. Res. Public Health.

[43] Barnes K., Newman E., Keenan G. (2023). A comparison of the impact of exposure to fit ideal and non-fit ideal body shapes in fitspiration imagery on women. Comput. Hum. Behav..

[44] Engel E., Gell S., Heiss R., Karsay K. (2024). Social media influencers and adolescents’ health: A scoping review of the research field. Soc. Sci. Med..

[45] Polivy J. (2001). The false hope syndrome: Unrealistic expectations of self-change. Int. J. Obes..

[46] Hawes T., Zimmer-Gembeck M.J., Campbell S.M. (2020). Unique associations of social media use and online appearance preoccupation with depression, anxiety, and appearance rejection sensitivity. Body Image.

[47] Wang W., Wang J., Liu Y., Deng L. (2025). Exploring the relationship between physical activity and social media addiction among adolescents through a moderated mediation model. Sci. Rep..

[48] Fulton S., Décarie-Spain L., Fioramonti X., Guiard B., Nakajima S. (2022). The menace of obesity to depression and anxiety prevalence. Trends Endocrinol. Metab..

[49] Gariepy G., Nitka D., Schmitz N. (2010). The association between obesity and anxiety disorders in the population: A systematic review and meta-analysis. Int. J. Obes..

[50] Haghighi M., Jahangard L., Ahmadpanah M., Bajoghli H., Holsboer-Trachsler E., Brand S. (2016). The relation between anxiety and BMI-is it all in our curves?. Psychiatry Res..

[51] Xiang L., Gou H., Hu C. (2025). The impact of weight self-stigma on appearance anxiety among female college students: A variable-centered and person-centered analysis. Front. Public Health.

[52] Abrante D., Carballeira M. (2023). Self-Exposure on Instagram and BMI: Relations With Body Image Among Both Genders. Eur. J. Psychol..

[53] Raspovic A., Prichard I., Salim A., Yager Z., Hart L. (2023). Body image profiles combining body shame, body appreciation and body mass index differentiate dietary restraint and exercise amount in women. Body Image.

[54] Li W., Ding H., Xu G., Yang J. (2023). The Impact of Fitness Influencers on a Social Media Platform on Exercise Intention during the COVID-19 Pandemic: The Role of Parasocial Relationships. Int. J. Environ. Res. Public Health.

[55] Cerolini S., Vacca M., Zegretti A., Zagaria A., Lombardo C. (2024). Body shaming and internalized weight bias as potential precursors of eating disorders in adolescents. Front. Psychol..

